# Supplementary material for: Type III secretion system effector YfiD inhibits the activation of host poly(ADP-ribose) polymerase-1 to promote bacterial infection
Source: Commun Biol. 2024 Feb 9;7:162. doi: 10.1038/s42003-024-05852-z (PMC10853565; doi:10.1038/s42003-024-05852-z)
Supplement: Supplementary file 1 — Supplementary Information [file 42003_2024_5852_MOESM1_ESM.pdf]

FIG S1

a

Protein	Coverage	MW (kDa)	Unique peptide	Description
TrxIP	42.4%	13.9	5	effector
EvpP	25.0%	20.6	3	T6SS effector
YfiD (ETAE_2732)	19.7%	14.2	3	Autonomous glycyl radical cofactor GrcA
EvpQ	8.67%	20.1	2	T6SS effector
EseG	4.0%	30.9	1	T3SS effector

b

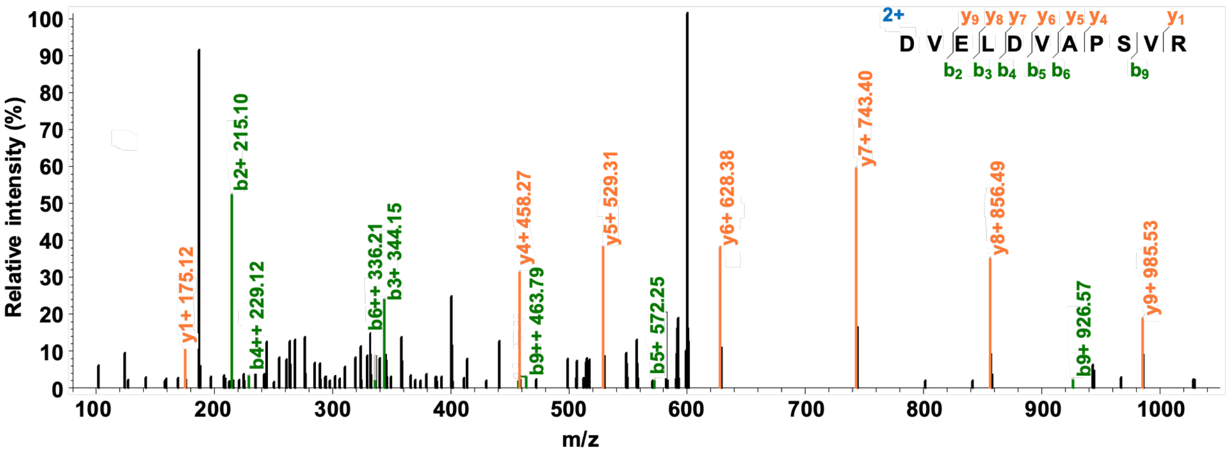

c

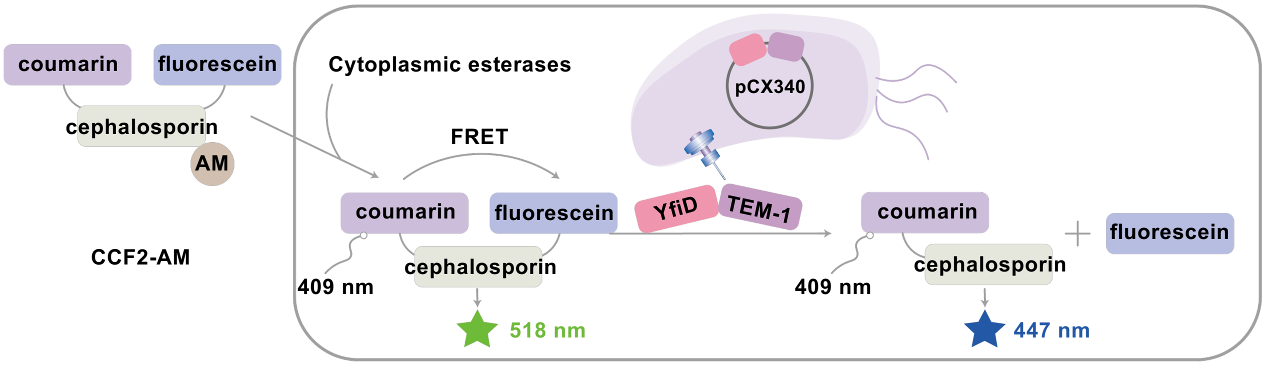

FIG S1

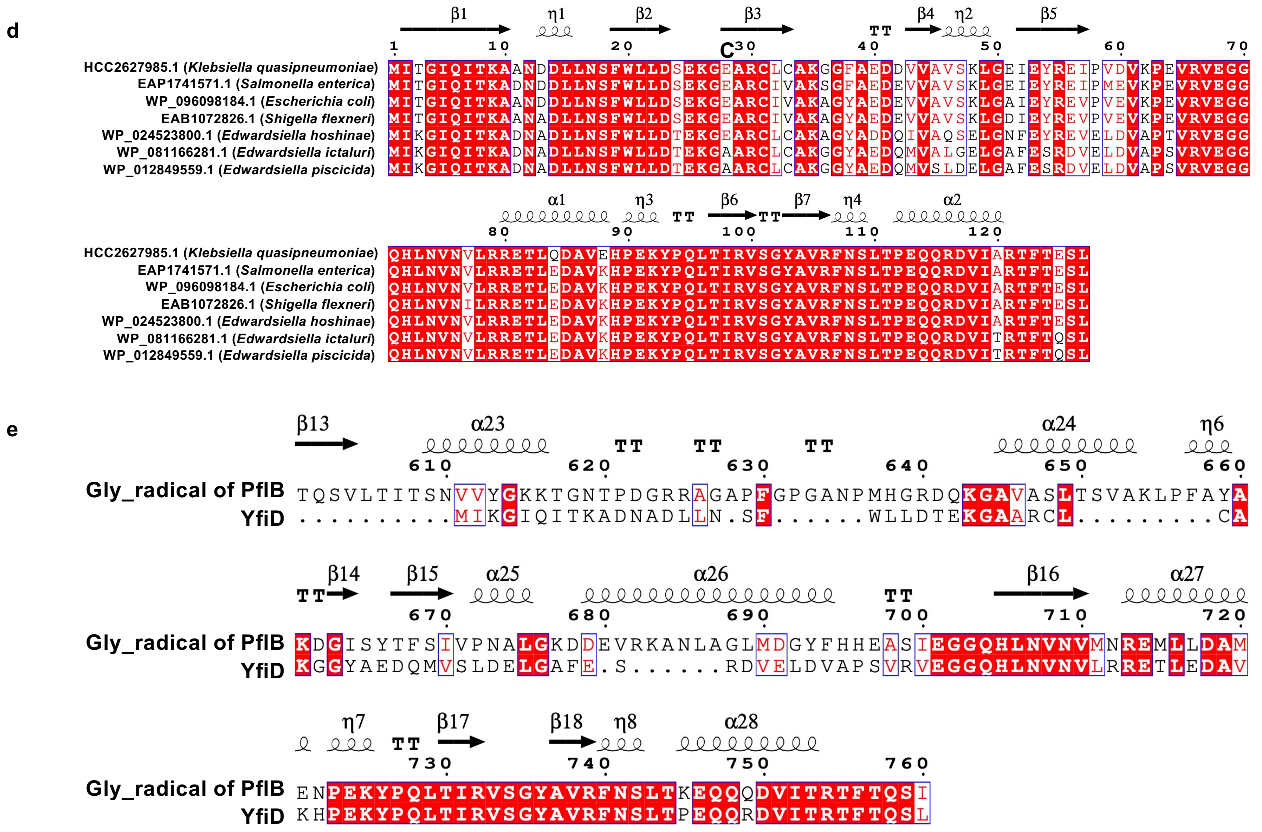

FIG S1 (**a**) Representative proteins identified by mass spectrometric analysis of the indicated band in Fig. 1a. (**b**) Mass spectrum of the peptide DVELDVAPSVR of YfiD obtained by mass spectrometric analysis of the indicated band in Fig. 1a. (**c**) Principle diagram of TEM-1β-lactamase system. FRET, fluorescence resonance energy transfer. TEM-1, TEM-1β-lactamase. AM, acetoxymethyl. (**d**) Multiple alignments of YfiD amino acid sequences in *Klebsiella*, *Salmonella*, *Shigella*, and *Edwardsiella*. (**e**) Sequence comparison between YfiD and the gly_radical domain of PflB in *E. piscicida*.

FIG S2

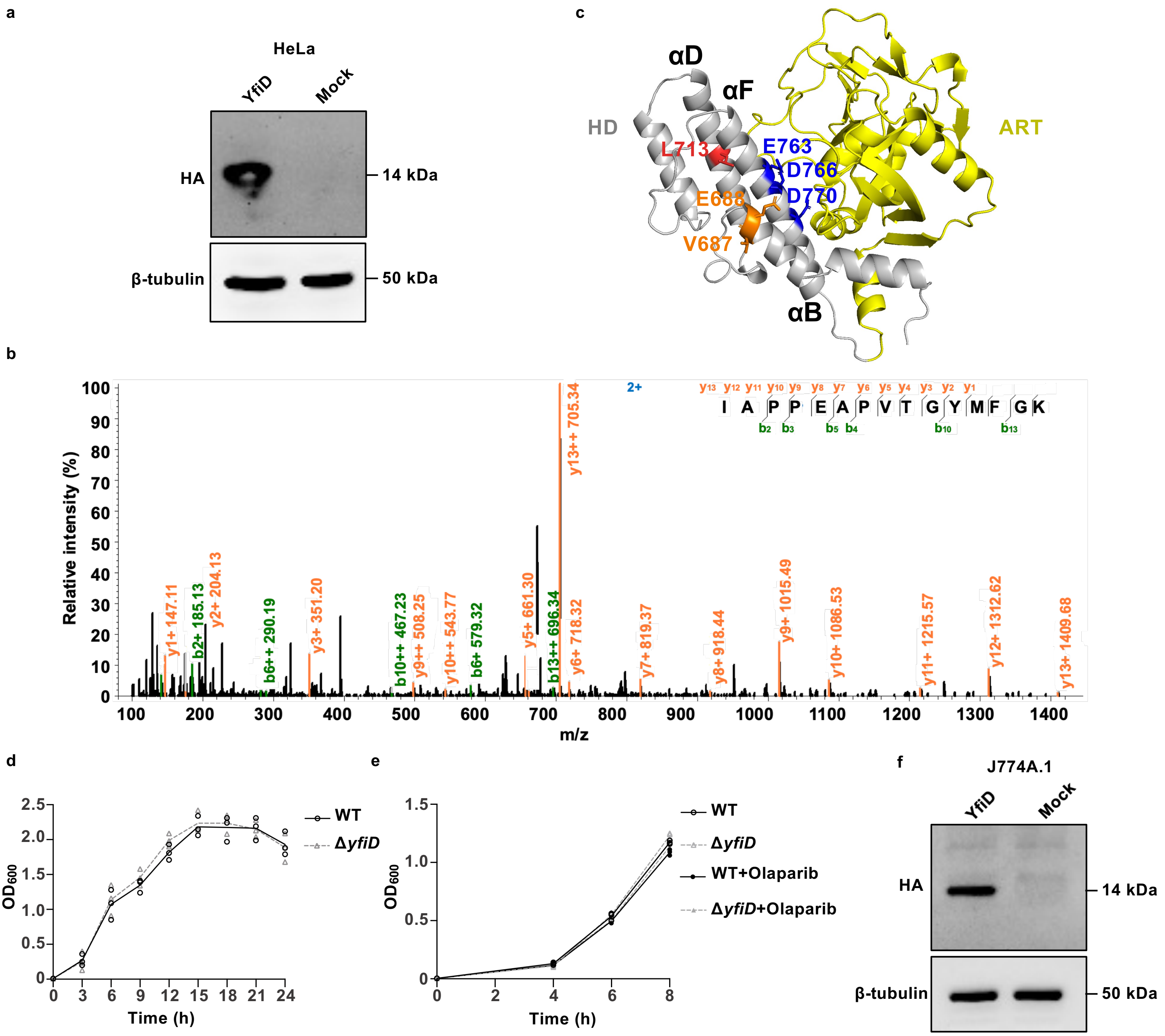

a
HeLa
YfiD
Mock
HA
14 kDa
β-tubulin
50 kDa
c
αD
αF
HD
ART
L713
E763
D766
D770
E688
V687
αB
b
Relative intensity (%)
m/z
y1+ 147.11
b2+ 185.13
y2+ 204.13
b6++ 290.19
y3+ 351.20
b10++ 467.23
y9++ 508.25
y10++ 543.77
b6+ 579.32
y5+ 661.30
b13++ 696.34
y13++ 705.34
y6+ 718.32
y7+ 819.37
y8+ 918.44
y9+ 1015.49
y10+ 1086.53
y11+ 1215.57
y12+ 1312.62
y13+ 1409.68
2+
I A P P E A P V T G Y M F G K
d
OD600
Time (h)
WT
ΔyfiD
e
WT
ΔyfiD
WT+Olaparib
ΔyfiD+Olaparib
f
J774A.1
YfiD
Mock
HA
14 kDa
β-tubulin
50 kDa

FIG S2

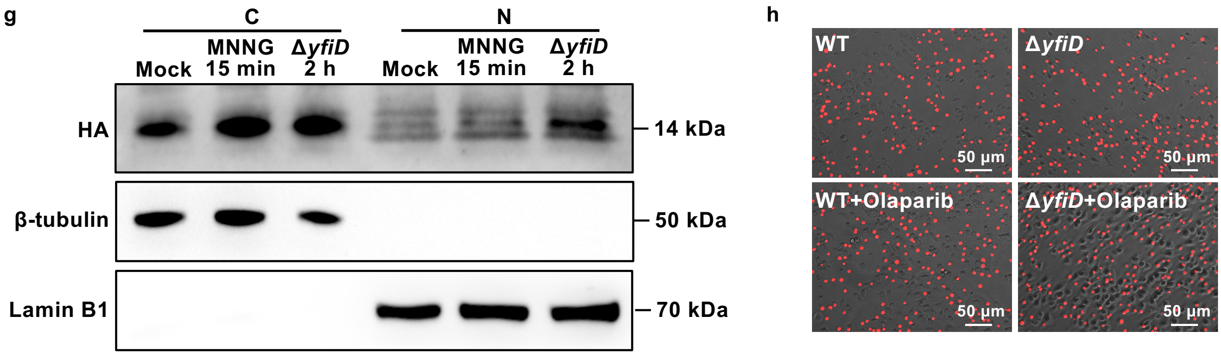

FIG S2 (**a**) HeLa cells stably expressing YfiD were verified by Western blot with anti-HA primary antibody. β-tubulin was served as a loading control. (**b**) Mass spectrum of the peptide IAPPEAPVTGYMFGK of PARP1 obtained by mass spectrometric analysis in Fig. 2a-b. (**c**) Structures of HD-ART domain of PARP1. The crystal structure of HD-ART showed the HD domain in gray and the ART domain in yellow. The crystal structure of HD-ART domain was predicted from Phyre2 and the molecular docking was monitored using PyMOL. V687 and E688 in αB (orange), L713 in αD (red), and E763, D766, and D770 in αF (blue) are highlighted. (**d-e**) Growth curves of *E. piscicida* WT and Δ*yfiD* in the presence or absence of Olaparib. WT and Δ*yfiD* cultured overnight were shaken in LB medium (d) or statically cultured in opti-MEM (e). OD_{600} values were measured at the indicated time. (**f**) J774A.1 cells stably expressing YfiD were verified by Western blot with anti-HA primary antibody. β-tubulin was served as a loading control. (**g**) Subcellular distributions of YfiD in J774A.1 cells expressing HA-tagged YfiD. The indicated cell was either exposed to 200 μM MNNG for 15 min or at 2 hours post-Δ*yfiD*-infection, respectively. The cytoplasmic and nuclear fractions were collected and analyzed with Western blot assay. β-tubulin and Lamin B1 were served as cytoplasmic and nuclear markers, respectively. (**h**) Cell viability of J774A.1 cells infected with *E. piscicida* WT or Δ*yfiD* was analyzed by PI uptake assay in the presence or absence of Olaparib. All images shown are representative of three independent experiments (n=3).

FIG S3

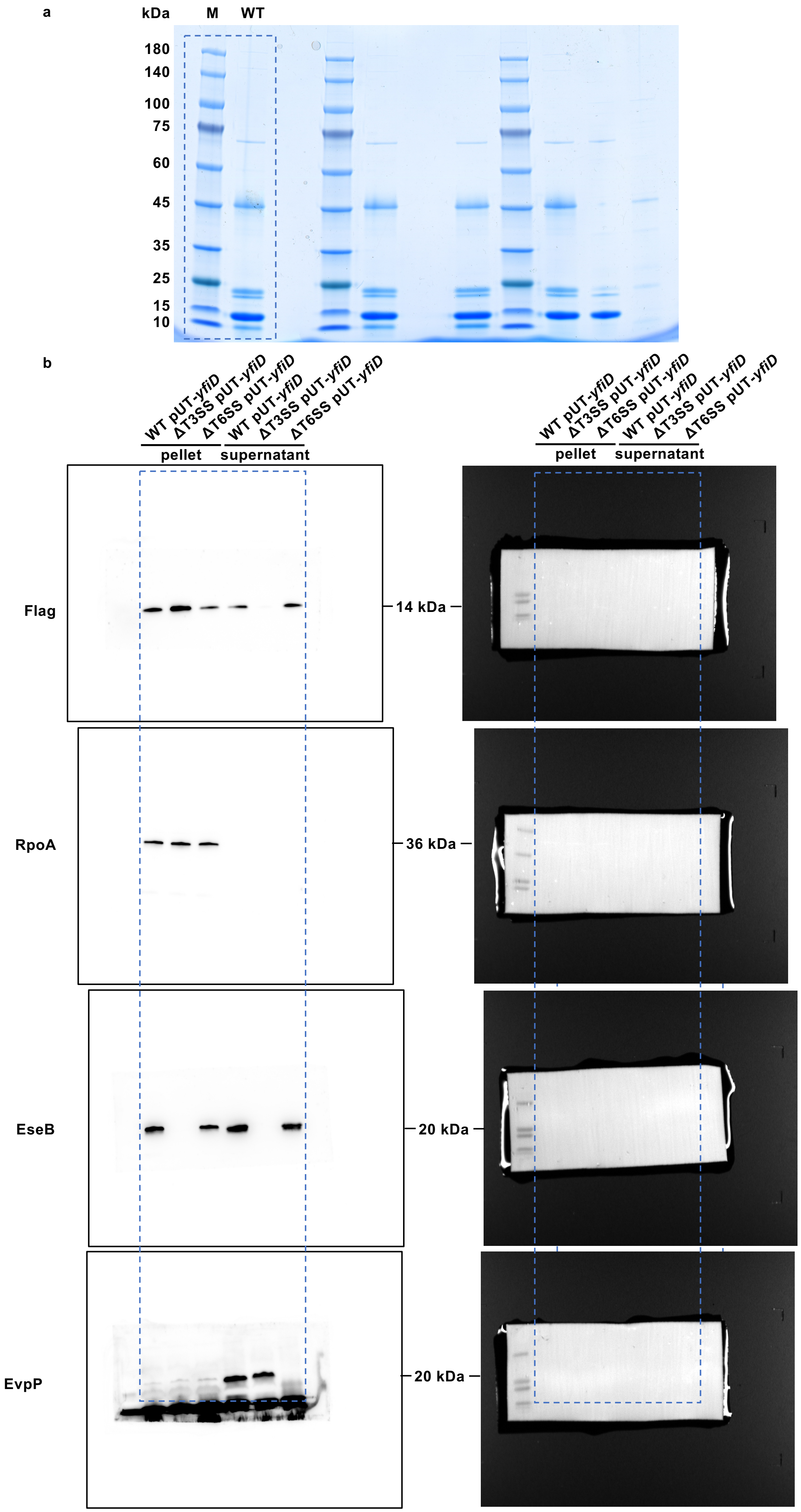
a
kDa
M
WT
180
140
100
75
60
45
35
25
15
10
b
WT pUT-yfiD
ΔT3SS pUT-yfiD
ΔT6SS pUT-yfiD
WT pUT-yfiD
ΔT3SS pUT-yfiD
ΔT6SS pUT-yfiD
pellet
supernatant
Flag
14 kDa
RpoA
36 kDa
EseB
20 kDa
EvpP
20 kDa

FIG S3

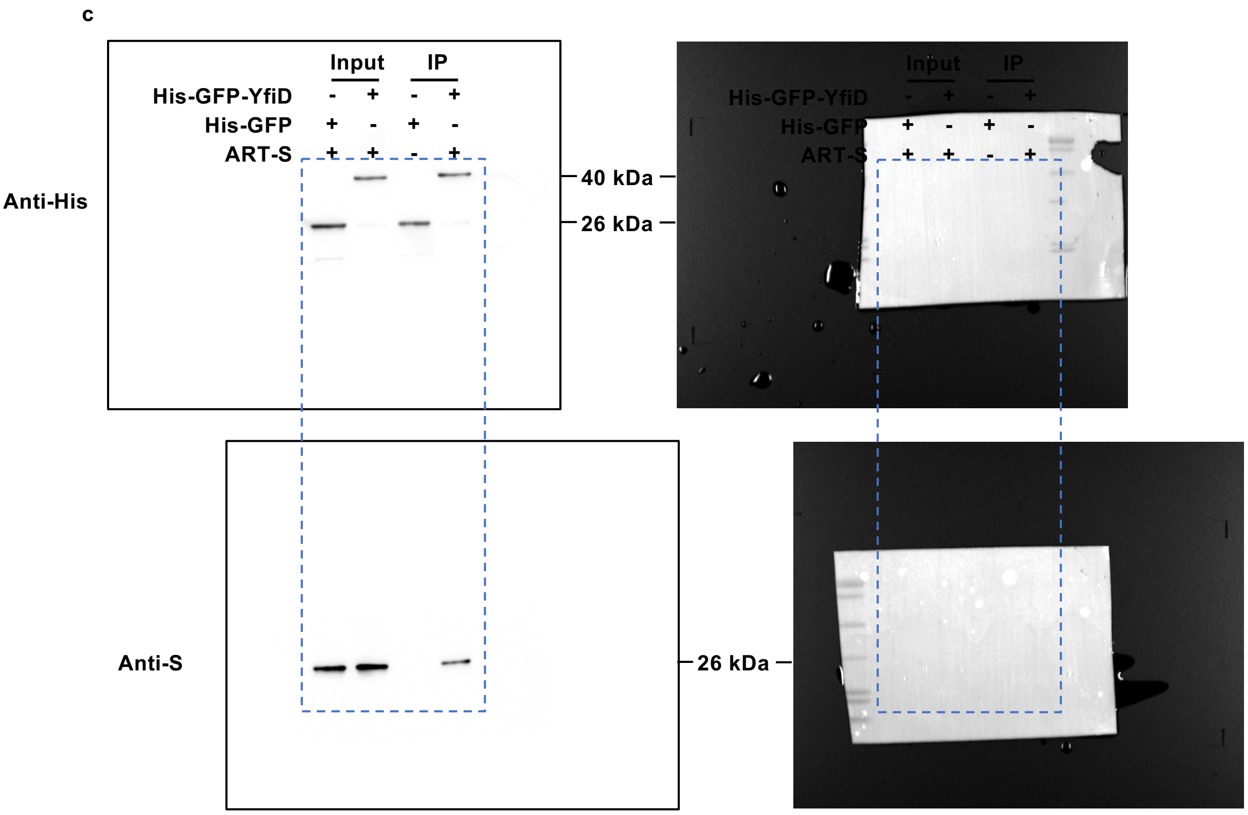
c
Input
IP
His-GFP-YfiD - + - +
His-GFP + - + -
ART-S + + - +
Anti-His
40 kDa
26 kDa
Anti-S
26 kDa

FIG S3

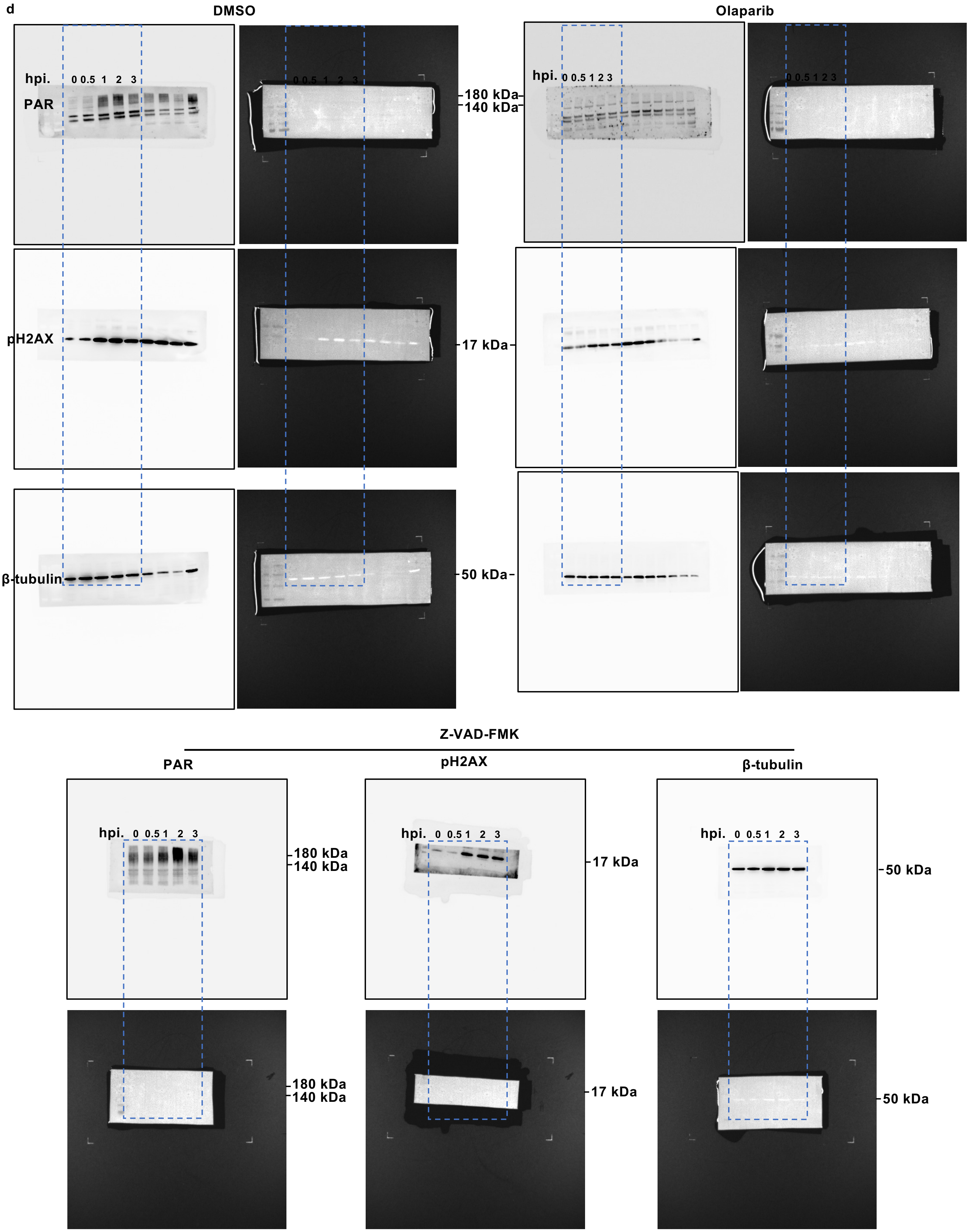
d
DMSO
Olaparib
hpi. 0 0.5 1 2 3
PAR
180 kDa
140 kDa
pH2AX
17 kDa
β-tubulin
50 kDa
Z-VAD-FMK
PAR
pH2AX
β-tubulin

FIG S3

e

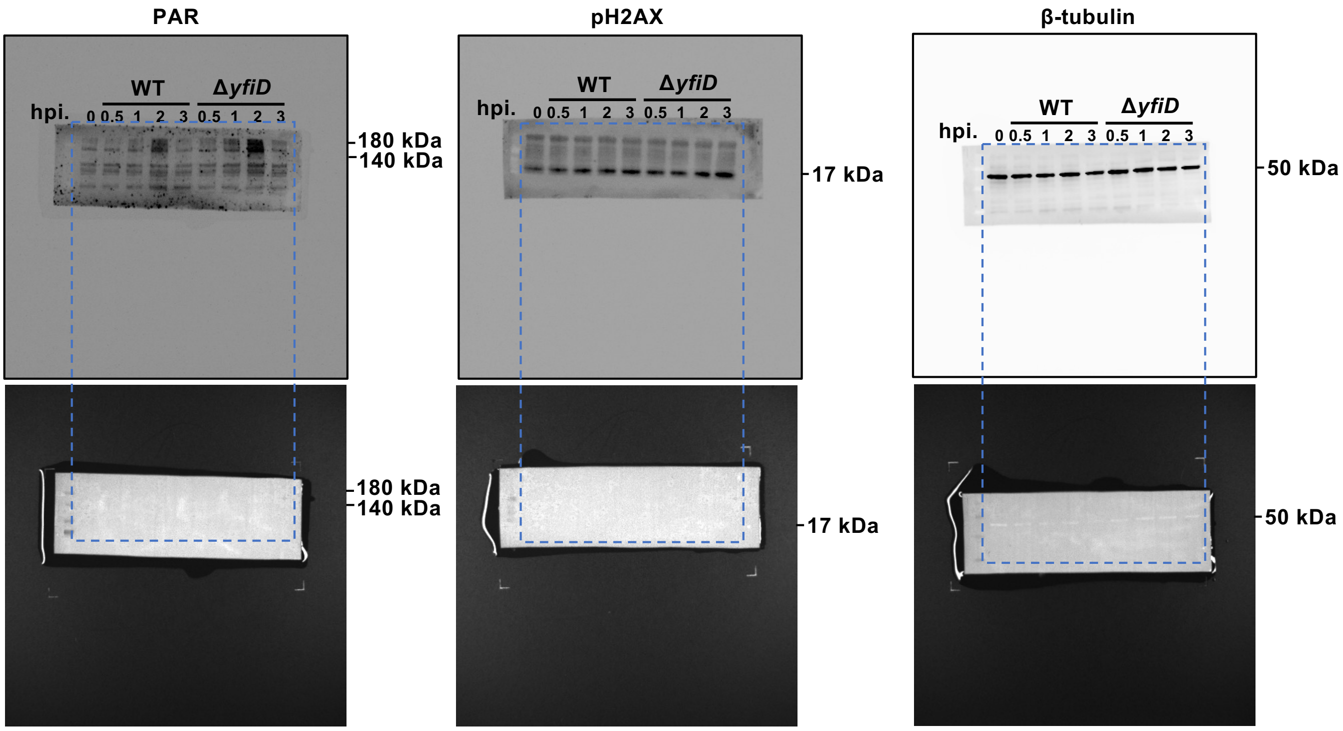
PAR
pH2AX
β-tubulin
WT
ΔyfiD
hpi.
0 0.5 1 2 3 0.5 1 2 3
180 kDa
140 kDa
17 kDa
50 kDa

f

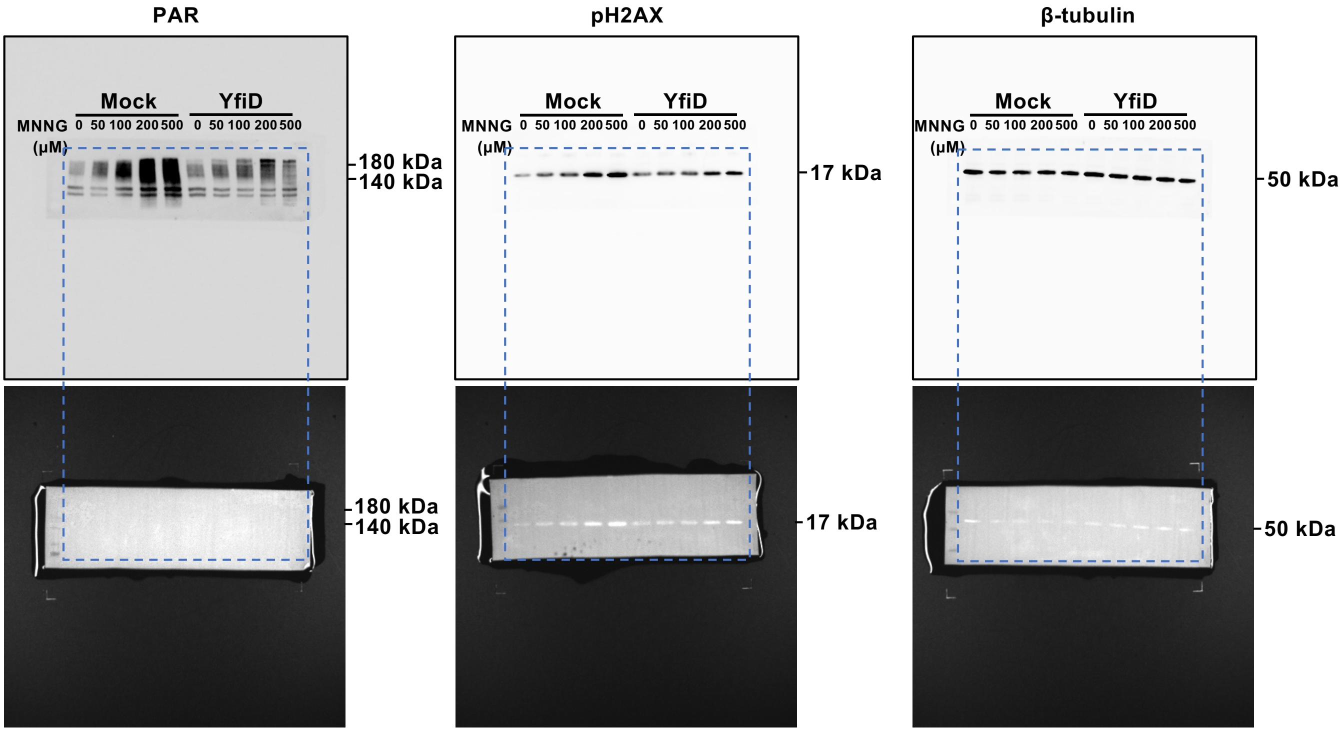
PAR
pH2AX
β-tubulin
Mock
YfiD
MNNG (μM)
0 50 100 200 500 0 50 100 200 500
180 kDa
140 kDa
17 kDa
50 kDa

FIG S3

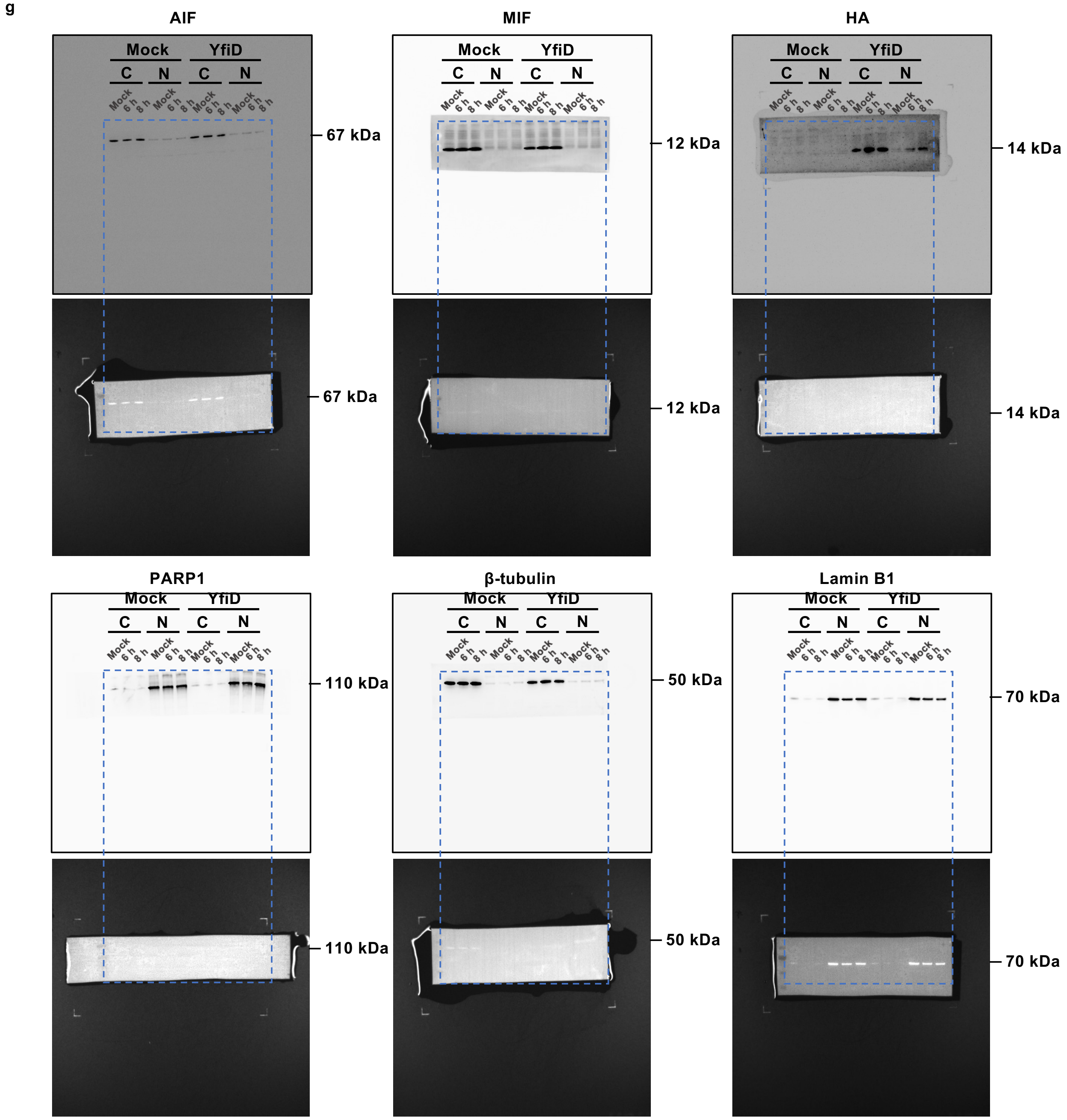
g
AIF
MIF
HA
Mock
YfiD
C
N
67 kDa
12 kDa
14 kDa
PARP1
β-tubulin
Lamin B1
110 kDa
50 kDa
70 kDa

FIG S3

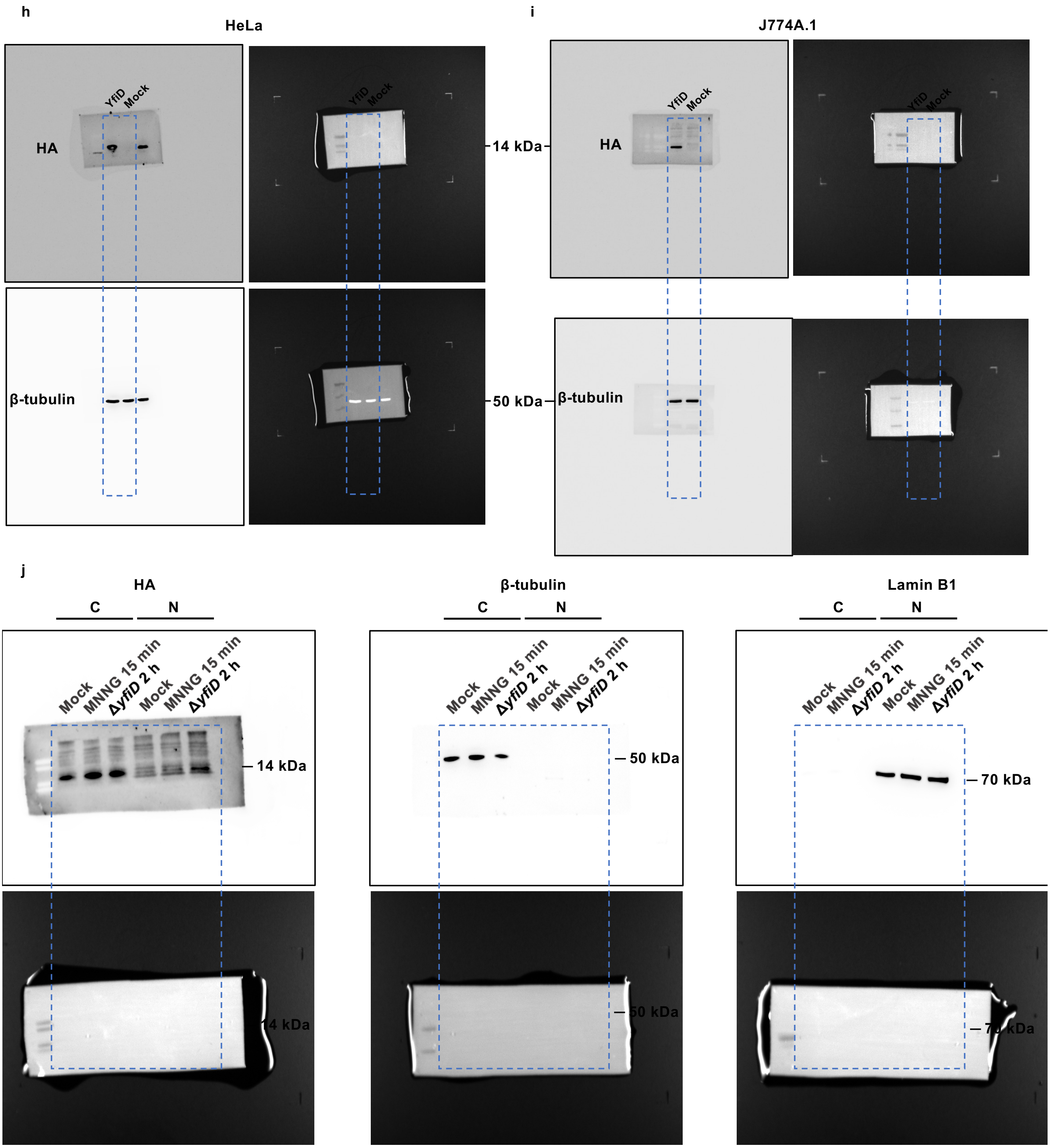

FIG S3 (**a**) The uncropped and unedited gel for Fig. 1a. (**b**) Uncropped and unedited blots for Fig. 1b. (**c**) Uncropped and unedited gels for Fig. 2f. (**d**) Uncropped and unedited gels for Fig. 3a. (**e**) Uncropped and unedited gels for Fig. 3b. (**f**) Uncropped and unedited gels for Fig. 3c. (**g**) Uncropped and unedited gels for Fig. 4c. (**h**) Uncropped and unedited gels for Fig. S2a. (**i**) Uncropped and unedited gels for Fig. S2f. (**j**) Uncropped and unedited gels for Fig. S2g.

Table S1 Strains or plasmids used in this study

Strain or plasmid	Relevant characteristics	Source or references
	Cells	
J774A.1	ATCC TIB-67	Lab collection
HeLa	ATCC No.CCL2	Lab collection
HEK293T	ATCC CRL-11268	Lab collection
	Strains	
E. coli		
SM10 λ*pir*	Host for π-requiring plasmids, conjugal donor	Lab collection
BL21(DE3)	Host strain for protein expression	Novagen
BTH101	Competent strain for BACTH system	
E. piscicida		
EIB202	Wild type, obtained from a mariculture farm in Shandong, China (CCTCC no. M 208068), Col^r	Lab collection
Δ*yfiD*	EIB202 Δ*yfiD*	This study
ΔT3SS	EIB202 Δ*eseB* Δ*eseC* Δ*eseD*	This study
ΔT6SS	EIB202 Δ*evpA* Δ*evpP*	This study
WT pUT-*yfiD*	EIB202/pUT-P_{0456}-*yfiD*-*flag*, Carb^r	This study
ΔT3SS pUT-*yfiD*	ΔT3SS/pUT-P_{0456}-*yfiD*-*flag*, Carb^r	This study
ΔT6SS pUT-*yfiD*	ΔT6SS/pUT-P_{0456}-*yfiD*-*flag*, Carb^r	This study
WT pCX340-*yfiD*	EIB202/pCX340-*yfiD*-TEM, Tet^r	This study
ΔT3SS pCX340-*yfiD*	ΔT3SS/pCX340-*yfiD*-TEM, Tet^r	This study
ΔT6SS pCX340-*yfiD*	ΔT6SS/pCX340-*yfiD*-TEM, Tet^r	This study
WT pCX340-*eseG*	EIB202/pCX340-*eseG*-TEM, Tet^r	Lab collection
ΔT3SS pCX340-*eseG*	ΔT3SS/pCX340-*eseG*-TEM, Tet^r	Lab collection
ΔT6SS pCX340-eseG	ΔT6SS/pCX340-*eseG*-TEM, Tet^r	Lab collection
Plasmids		
pDM4	Suicide vector, *pir* dependent, R6K, SacBR, [illegible]	KC795686 (NCBI)
pUT	Derived from pUC18 with the deletion of *lac* promoter, Carb^r	Xiao et al., 2011[1]
pCDH	A vector for lentiviral integration, Carb^r	Chen et al., 2022[2]
pCMV-VSVG	Expressing VSV-G for lentiviral packaging, Carb^r	Beyotime
pCAG-dR8.9	Expressing GAG and POL for lentiviral [illegible]	Beyotime

Table S1 Strains or plasmids used in this study

pET28a	A vector for the expression of C-His-tagged protein, Km^r	Novagen
pETDuet-1	A vector for the expression of N-His-tagged protein, Carb^r	Novagen
pCX340	A vector containing TEM-1, Tet^r	Charpentier et al., 2004[3]
pKT25	pKT carrying T25 fragment of CyaA for BACTH	Euromedex
pUT18	pUT carrying T18 fragment of CyaA for BACTH	Euromedex
pET28a-*hns*	pET28a carrying *hns*, Km^r	This study
pET28a-*yfiD*	pET28a carrying *yfiD*, Km^r	This study
pDM4-*yfiD*	pDM4 with *yfiD* deleted from nt 4 to 381, Cm^r	This study
pCDH-*yfiD*-HA	pCDH carrying HA-tagged *yfiD*, Carb^r	This study
pETDuet-1-GFP	pETDuet-1 carrying *his-gfp*, Carb^r	This study
pETDuet-1-GFP-YfiD	pETDuet-1 carrying *his-gfp-yfiD*, Carb^r	This study
pETDuet-1-ART	pETDuet-1 carrying ART-S, Carb^r	This study
pCX340-*yfiD*	pCX340 carrying *yfiD*, Tet^r	This study
pUT-*yfiD*	pUT carrying *yfiD*, Carb^r	This study
pKT25-ART	pKT25 carrying ART, Km^r	This study
pKT25-HD-ART	pKT25 carrying HD-ART, Km^r	This study
pUT18-YfiD	pUT18 carrying YfiD, Carb^r	This study

Table S2 Primers used in this study

Primer name	Primer sequence (5’ to 3’)
yfiD-flag-F	tttatcgaggtgagaggcacatgattaaaggtattcagatcaccaaggctgaca
yfiD-flag-R	tagtctgagccaccgccacccaggctctgggtgaaggtacgg
yfiD-P1	gagctcaggttacccgcatgcaagatctatcgctcgacggtcagtacggtg
yfiD-P2	ccgccccttacatttgttgcctccaattgagatagagcaaaaatc
yfiD-P3	gcaacaaatgtaaggggcggcggcgcgag
yfiD-P4	ccctcgagtacgcgtcactagtggggccctccggatatcagccacgtgttcaac
yfiD-TEM-F	ataaggaggaataacatatgatgattaaaggtattcagatcaccaaggctgaca
yfiD-TEM-R	gaattctccgcggaggtacccaggctctgggtgaaggtacgg
pCDH-*yfiD*-F	agaagattctagagctagcgaattcatgattaaaggtattcagatcaccaaggctgaca
pCDH-*yfiD*-R1	aacatcgtatgggtagcctccgcctccgcctcccaggctctgggtgaaggtacgg
pCDH-*yfiD*-R2	atcgcagatccttcgcggccgcttaagcgtaatctggaacatcgtatgggtagcctccgcct
pUT18-yfiD-F	gcatgcctgcaggtcgactctagagatgattaaaggtattcagatcaccaaggct
pUT18-yfiD-R	ccgtggcctcgctggcggctgaattcaggctctgggtgaaggtacgg
pKT25-F1F1-F	gcgggctgcagggtcgactctagaggggtccggatcgggtatggcggaggcctcggag
pKT25-F1F1-R	ttgtaaaacgacggccagtgaattc ttaaccttttctctttccttcattcttgatggcc
pKT25-F3-F	gcgggctgcagggtcgactctagaggggtccggatcgggtgacgaggtggatggaacagatgaagtg
pKT25-F3-R	ttgtaaaacgacggccagtgaattcttacagtgccagtggtgctggg
pKT25-BRCT-F	gcgggctgcagggtcgactctagaggggtccggatcgggtcccctctctgtcacctcagcacc
pKT25-BRCT-R	ttgtaaaacgacggccagtgaattc ttatgacttccccttgggggccac
pKT25-WGR-F	gcgggctgcagggtcgactctagaggggtccggatcgggtgctgcaccctccaagaagagc
pKT25-WGR-R	ttgtaaaacgacggccagtgaattc ttactcttcgtcctggccatagtcaatctc
pKT25-HD-F	gcgggctgcagggtcgactctagaggggtccggatcgggtaagtcgaagctgccgaagcc
pKT25-HD-R	ttgtaaaacgacggccagtgaattcttagctgtcgtcagagccacccc
pKT25-ART-F	gcgggctgcagggtcgactctagaggggtccggatcgggtagcaaggatcccatcgacgtcaac
pKT25-ART-R	ttgtaaaacgacggccagtgaattcttaccacagggatgtcttaaaattgaacttgagtttcag
IL-1α-F	acgtcaagcaacgggaagat
IL-1α-R	aaggtgctgatctgggttgg
IL-1β-F	tgccaccttttgacagtgatg
IL-1β-R	aaaggtttggaagcagccct
IL-6-F	ccccaatttccaatgctctcc
IL-6-R	tgccgagtagatctcaaagtga

Supplementary References

1. Xiao, Y., Liu, Q., Chen, H. & Zhang, Y. X. A stable plasmid system for heterologous antigen expression in attenuated *Vibrio anguillarum*. *Vaccine*. **29**, 6986-6993 (2011).
2. Chen, C. et al. ATF4-dependent fructolysis fuels growth of glioblastoma multiforme. *Nat Commun*. 13, 6108 (2022).
3. Charpentier, X. & Oswald, E. Identification of the secretion and translocation domain of the enteropathogenic and enterohemorrhagic *Escherichia coli* effector Cif, using TEM-1 beta-lactamase as a new fluorescence-based reporter. *J Bacteriol*. 186, 5486-5495 (2004).
