# Supplementary material for: Type III secretion system effector YfiD inhibits the activation of host poly(ADP-ribose) polymerase-1 to promote bacterial infection
Source: Commun Biol. 2024 Feb 9;7:162. doi: 10.1038/s42003-024-05852-z (PMC10853565; doi:10.1038/s42003-024-05852-z)
Supplement: Supplementary file 2 — Supplementary Data 1 [file 42003_2024_5852_MOESM2_ESM.pdf]

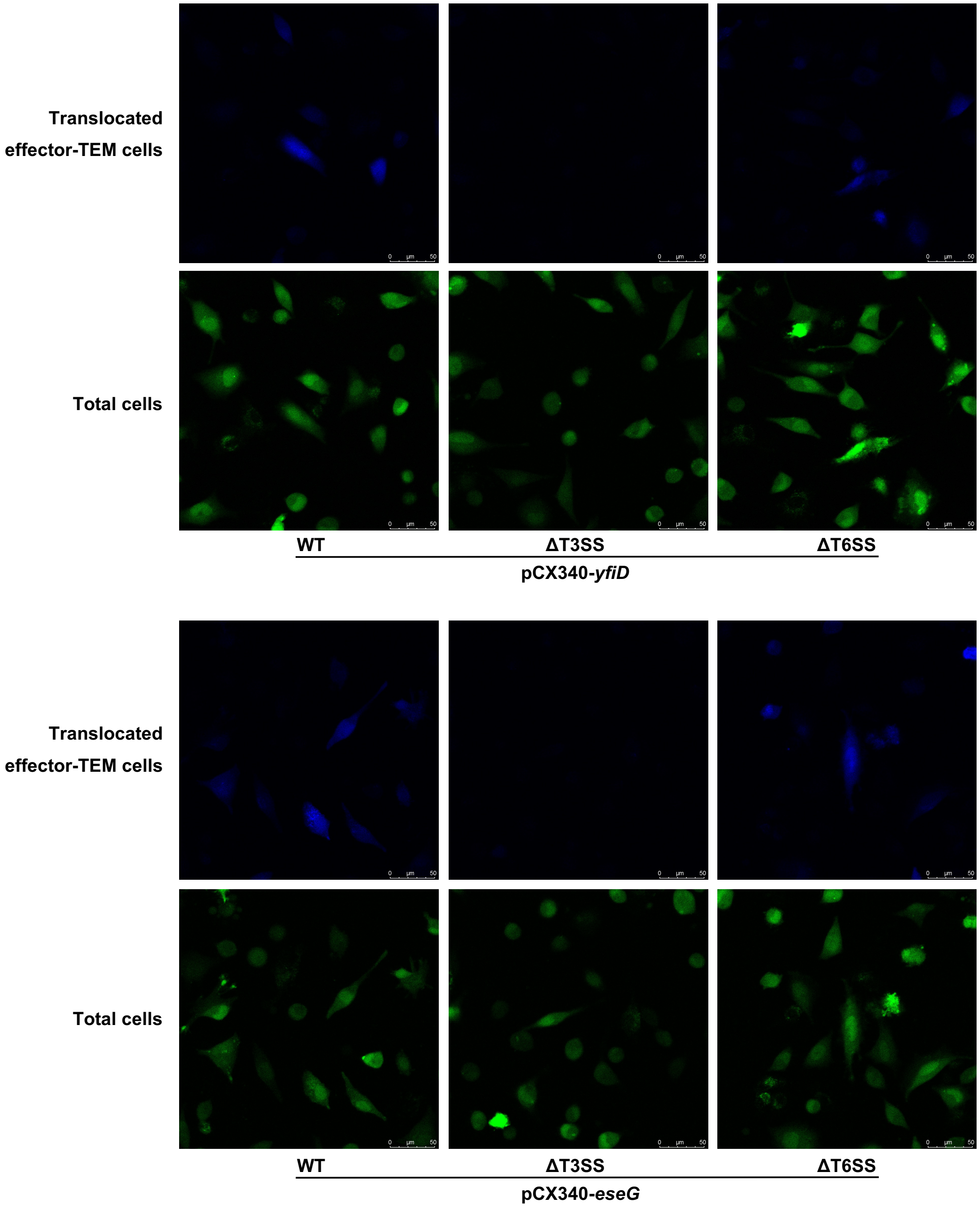
Translocated effector-TEM cells
Total cells
WT
ΔT3SS
ΔT6SS
pCX340-*yfiD*
Translocated effector-TEM cells
Total cells
WT
ΔT3SS
ΔT6SS
pCX340-*eseG*

Fig. 1c

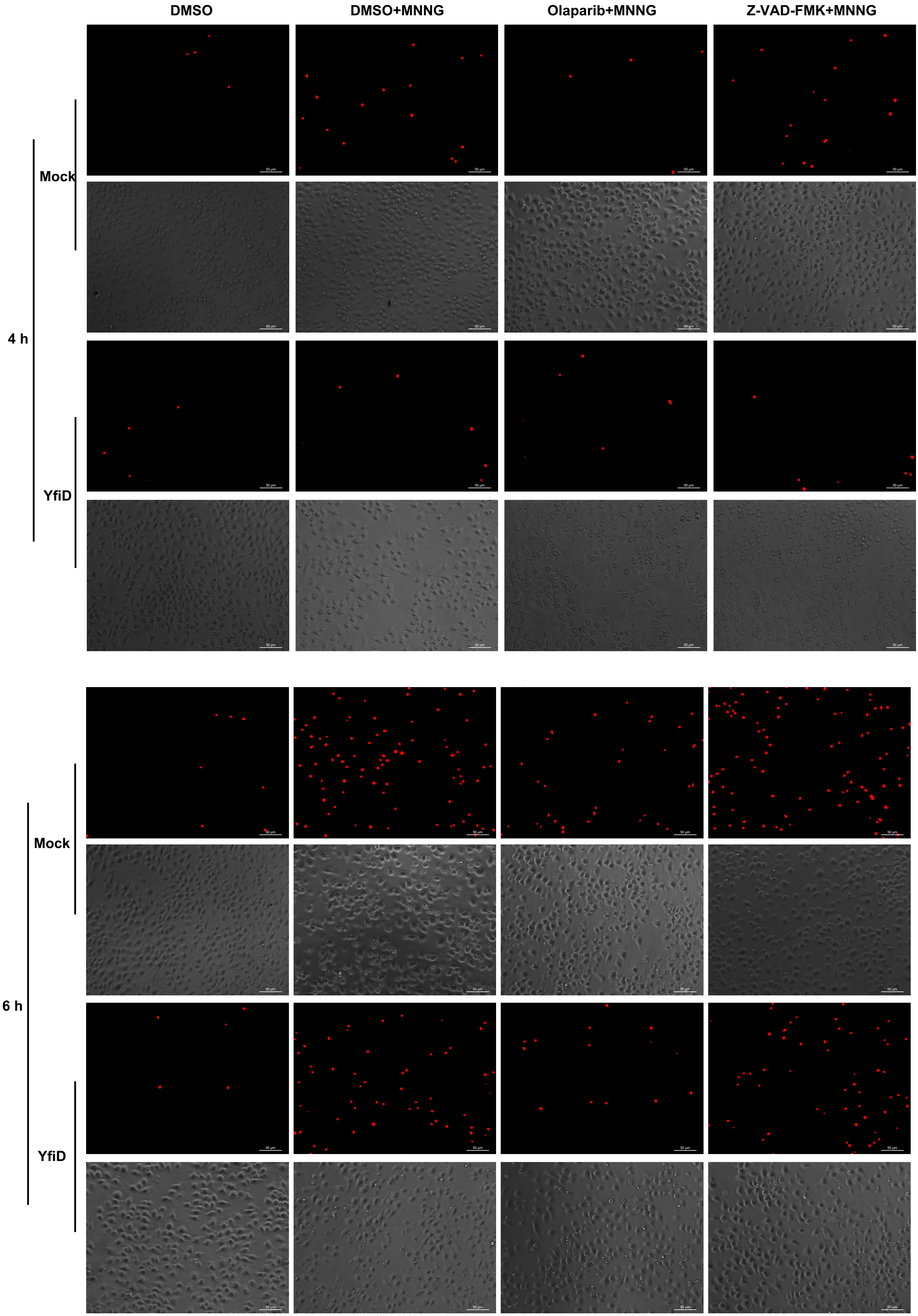
DMSO
DMSO+MNNG
Olaparib+MNNG
Z-VAD-FMK+MNNG
4 h
Mock
YfiD
6 h
Mock
YfiD

Fig. 4a

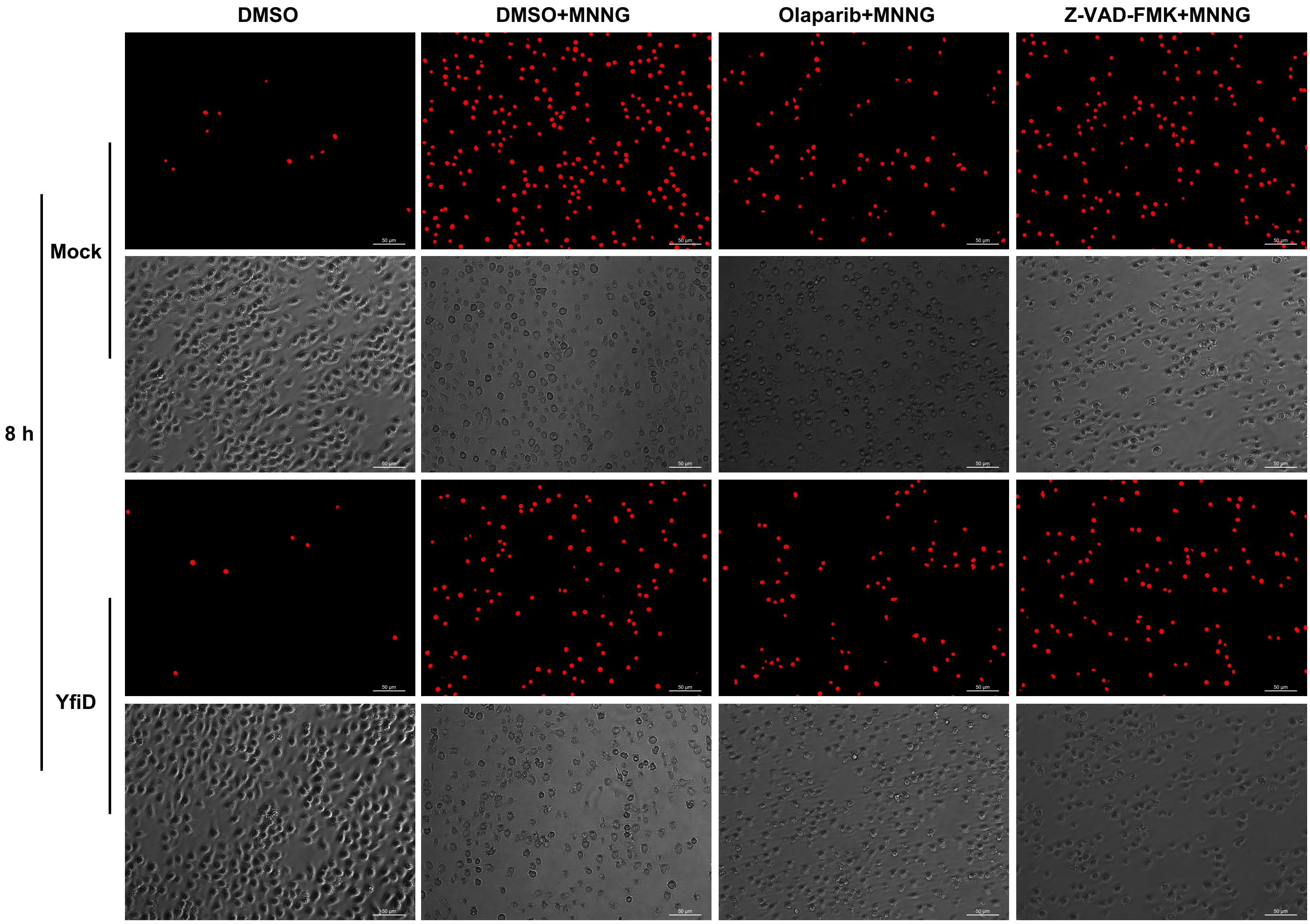

Fig. 4a

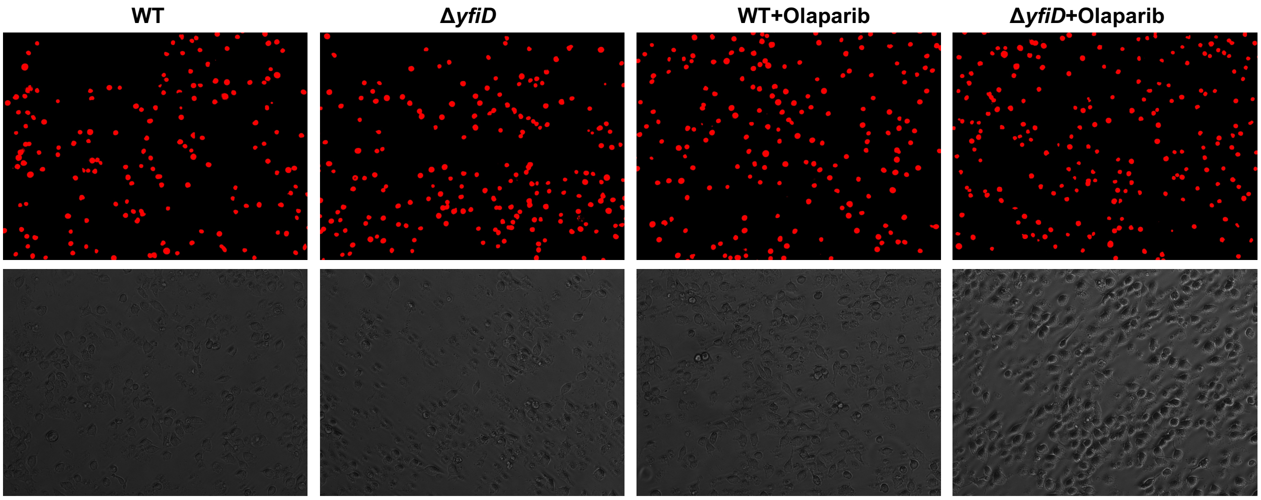

Fig. S2h
