# Supplementary material for: Type III secretion system effector YfiD inhibits the activation of host poly(ADP-ribose) polymerase-1 to promote bacterial infection
Source: Commun Biol. 2024 Feb 9;7:162. doi: 10.1038/s42003-024-05852-z (PMC10853565; doi:10.1038/s42003-024-05852-z)
Supplement: Supplementary file 3 — Supplementary Data 2 [file 42003_2024_5852_MOESM3_ESM.pdf]

WT pCX340 -*yfiD*	ΔT3SS pCX340 -*yfiD*	ΔT6SS pCX340 -*yfiD*	WT pCX340 -*eseG*	ΔT3SS pCX340 -*eseG*	ΔT6SS pCX340 -*eseG*
13.3	0	13	17.9	0	23.1
14.8	0	18.2	21.7	2.5	25
20	3.8	20	23.8	3.2	26.1

Fig. 1d

	Zip	Zip	-	Full	F1F2	F3	BRCT	WGR	HD	ART	HD-ART	ART	ART
	Zip	YfiD	YfiD	YfiD	YfiD	YfiD	YfiD	YfiD	YfiD	YfiD	YfiD	Zip	-
A420	2.317	0.193	0.19	0.17	0.18	0.191	0.203	0.179	0.166	1.367	0.196	0.187	0.202
	2.428	0.191	0.189	0.177	0.172	0.172	0.166	0.177	0.172	1.321	0.202	0.193	0.181
	2.334	0.165	0.197	0.188	0.174	0.197	0.194	0.168	0.174	1.38	0.202	0.183	0.204
	Zip	Zip	-	Full	F1F2	F3	BRCT	WGR	HD	ART	HD-ART	ART	ART
	Zip	YfiD	YfiD	YfiD	YfiD	YfiD	YfiD	YfiD	YfiD	YfiD	YfiD	Zip	-
OD600	0.81	0.72	0.73	0.81	0.76	0.89	0.82	0.84	0.86	0.81	0.77	0.84	0.78
	0.73	0.88	0.84	0.77	0.78	0.85	0.88	0.78	0.84	0.87	0.73	0.87	0.83
	0.75	0.84	0.86	0.9	0.86	0.93	0.92	0.89	0.8	0.95	0.76	0.93	0.85
t/min	8	20	20	20	20	20	20	20	20	20	20	20	20
	Zip	Zip	-	Full	F1F2	F3	BRCT	WGR	HD	ART	HD-ART	ART	ART
	Zip	YfiD	YfiD	YfiD	YfiD	YfiD	YfiD	YfiD	YfiD	YfiD	YfiD	Zip	-
**tosidase**	1787.8	67.0	65.1	52.5	59.2	53.7	61.9	53.3	48.3	421.9	63.6	55.7	64.7
	2078.8	54.3	56.3	57.5	55.1	50.6	47.2	56.7	51.2	379.6	69.2	55.5	54.5
	1945.0	49.1	57.3	52.2	50.6	53.0	52.7	47.2	54.4	363.2	66.4	49.2	60.0

Fig. 2e

	Zip	Zip	-	WT	ΔVE	763/770/776A	L713F	Combined	Combined	Combined
	Zip	YfiD	YfiD	YfiD	YfiD	YfiD	YfiD	YfiD	Zip	-
A420	2.348	0.187	0.217	0.239	0.18	0.266	0.234	1.328	0.175	0.192
	2.41	0.212	0.212	0.257	0.21	0.256	0.218	1.241	0.239	0.214
	2.351	0.225	0.221	0.218	0.215	0.179	0.231	1.314	0.215	0.25
	Zip	Zip	-	WT	ΔVE	763/770/776A	L713F	Combined	Combined	Combined
	Zip	YfiD	YfiD	YfiD	YfiD	YfiD	YfiD	YfiD	Zip	-
OD600	0.83	0.79	0.94	0.71	0.75	0.73	0.82	0.79	0.7	0.76
	0.78	0.83	0.84	0.74	0.78	0.71	0.76	0.83	0.81	0.81
	0.81	0.8	0.8	0.79	0.71	0.69	0.74	0.87	0.78	0.8
t/min	8	25	25	25	25	25	25	25	25	25
	Zip	Zip	-	WT	ΔVE	763/770/776A	L713F	Combined	Combined	Combined
	Zip	YfiD	YfiD	YfiD	YfiD	YfiD	YfiD	YfiD	Zip	-
**β-galactosidase activity**	1768.1	47.3	46.2	67.3	48.0	72.9	57.1	336.2	50.0	50.5
	1931.1	51.1	50.5	69.5	53.8	72.1	57.4	299.0	59.0	52.8
	1814.0	56.3	55.3	55.2	60.6	51.9	62.4	302.1	55.1	62.5

Fig. 2h

	PARP1	PARP1+Olaparib	PARP1+YfiD	PARP1+H-NS	PARP1+1604
Values	19186	3434	7674	19570	18092
	16713	3443	7939	15309	15141
	19074	3624	8641	16823	18521

	PARP1	PARP1+Olaparib	PARP1+YfiD	PARP1+H-NS	PARP1+1604
**Relative values**	100	17.9	40.0	102.0	94.3
	100	20.6	47.5	91.6	90.6
	100	19.0	45.3	88.2	97.1

Fig. 2i

		HA			YfiD		
4 h	DMSO	0.82	0.87	1.1	0.89	1.2	1.3
	MNNG	2.4	3.8	3.9	1.7	2.1	2.1
	Olaparib+MNNG	0.97	1.56	1.82	1.02	1.58	1.81
	V-ZAD-FMK+MNNG	2.7	3.4	3.8	1.2	1.9	2.5
6 h	DMSO	1.1	1.4	1.5	0.93	1.1	1.3
	MNNG	24.6	27.2	32.8	16.4	18.3	20.2
	Olaparib+MNNG	7.62	8.12	8.62	5.51	7.39	7.85
	V-ZAD-FMK+MNNG	23.3	26.2	28.9	16.9	17.7	22.1
8 h	DMSO	1.25	2.2	2.52	1.67	1.98	2.45
	MNNG	52.4	56.1	60.2	37.9	42.4	43.5
	Olaparib+MNNG	22.3	25.1	27.3	21.2	22.9	23.9
	V-ZAD-FMK+MNNG	53.2	58	59.1	40.5	44.7	45.8

Fig. 4b

	Mock			WT			ΔyfiD			Olaparib Mock			Olaparib WT			Olaparib Δ*yfiD*		
IL-1α	1	1	1	32.8	32.5	36.1	90.6	85.3	107	0.85	1.19	0.769	33.8	29.4	38.4	33.1	34.8	41.2
IL-1β	1	1	1	181.7	172.3	169.4	481.3	409.9	435.6	1.11	0.693	0.634	194.5	161.6	176.4	151.9	140.7	156.5
IL-6	1	1	1	40.1	59.1	70.6	77.6	123.6	159	0.45	1.16	0.711	33.6	44.5	53.6	34	52.2	63.9

Fig. 5a

WT			Δ*yfiD*			WT+Olaparib			Δ*yfiD* +Olaparib		
49.6	49.8	61	65.8	70.5	72.3	45.5	47.1	57.1	47.9	51.2	56.7

Fig. 5b

WT	Δ*yfiD*	WT+Olaparib	Δ*yfiD*+Olaparib
5.4	2.2	9	7.2
7.6	4	11.2	10.4
5	2.8	8	8.8

Fig. 5c

Liver				Spleen				Kidney			
CFU/g		**lg(CFU/g)**		CFU/g		**lg(CFU/g)**		CFU/g		**lg(CFU/g)**	
WT	Δ*yfiD*	WT	Δ*yfiD*	WT	Δ*yfiD*	WT	Δ*yfiD*	WT	Δ*yfiD*	WT	Δ*yfiD*
1520000	100000	6.18	5.00	1450000	63000	6.16	4.80	1610000	26900	6.21	4.43
1800000	300000	6.26	5.48	183000	106000	5.26	5.03	780000	40000	5.89	4.60
2400000	63000	6.38	4.80	1750000	190000	6.24	5.28	1460000	100000	6.16	5.00
2600000	40000	6.41	4.60	250000	300000	5.40	5.48	940000	23600	5.97	4.37
2800000	31800	6.45	4.50	260000	238000	5.41	5.38	116000	126000	5.06	5.10
286000	160000	5.46	5.20	400000	185000	5.60	5.27	124000	80000	5.09	4.90
520000	158000	5.72	5.20	475000	65000	5.68	4.81	140000	95000	5.15	4.98
600000	151000	5.78	5.18	500000	200000	5.70	5.30	260000	138000	5.41	5.14
660000	26600	5.82	4.42	318000	164000	5.50	5.21	320000	14000	5.51	4.15
480000	140000	5.68	5.15	2100000	27800	6.32	4.44	380000	25200	5.58	4.40

Fig. 5d

	PBS	WT	Δ*yfiD*
1 d (Mortality)	0	0	0
2 d (Mortality)	0	0	0
3 d (Mortality)	0	2	4
4 d (Mortality)	0	8	4
5 d (Mortality)	0	8	3
6 d (Mortality)	0	2	1
7 d (Survival)	20	0	8

**Percent survival**	PBS	WT	Δ*yfiD*
1 d	100	100	100
2 d	100	100	100
3 d	100	90	80
4 d	100	50	60
5 d	100	10	45
6 d	100	0	40

Fig. 5e

Time/h		WT			ΔyfiD	
0	0.01	0.01	0.01	0.01	0.01	0.01
3	0.25	0.2	0.36	0.23	0.13	0.4
6	1.09	0.85	1.28	1.16	0.91	1.35
9	1.39	1.24	1.41	1.45	1.34	1.58
12	1.93	1.71	1.81	2.09	1.9	1.98
15	2.34	2.06	2.15	2.42	2.15	2.14
18	2.31	1.97	2.24	2.35	2.08	2.28
21	2.31	1.99	2.19	2.28	2.04	2.13
24	2.12	1.88	1.79	2.09	1.89	1.68

Fig. S2d

Time/h	WT			Δ*yfiD*			WT+Olaparib			Δ*yfiD* +Olaparib		
0	0.004	0.004	0.004	0.004	0.004	0.004	0.004	0.004	0.004	0.004	0.004	0.004
4	0.14	0.13	0.12	0.11	0.1	0.12	0.12	0.13	0.1	0.12	0.13	0.14
6	0.56	0.55	0.5	0.57	0.56	0.52	0.48	0.53	0.48	0.51	0.49	0.5
8	1.19	1.17	1.16	1.2	1.25	1.24	1.06	1.09	1.11	1.13	1.16	1.12

Fig. S2e
