# Supplementary material for: Type III secretion system effector YfiD inhibits the activation of host poly(ADP-ribose) polymerase-1 to promote bacterial infection
Source: Commun Biol. 2024 Feb 9;7:162. doi: 10.1038/s42003-024-05852-z (PMC10853565; doi:10.1038/s42003-024-05852-z)
Supplement: Supplementary file 4 — Description of Additional Supplementary Files [file 42003_2024_5852_MOESM4_ESM.pdf]

Description of Additional Supplementary Files

File name: Supplementary Data 1
Description: Uncropped and unedited blots and gels.

File name: Supplementary Data 2
Description: Data points of statistical images.
