# Supplementary material for: Type III secretion system effector YfiD inhibits the activation of host poly(ADP-ribose) polymerase-1 to promote bacterial infection
Source: Commun Biol. 2024 Feb 9;7:162. doi: 10.1038/s42003-024-05852-z (PMC10853565; doi:10.1038/s42003-024-05852-z)
Supplement: Supplementary file 6 — Reporting Summary [file 42003_2024_5852_MOESM6_ESM.pdf]

nature portfolio

Corresponding author(s): Shuai Shao

Last updated by author(s): Jan 9, 2024

Reporting Summary

Nature Portfolio wishes to improve the reproducibility of the work that we publish. This form provides structure for consistency and transparency in reporting. For further information on Nature Portfolio policies, see our Editorial Policies and the Editorial Policy Checklist.

Statistics

For all statistical analyses, confirm that the following items are present in the figure legend, table legend, main text, or Methods section.

n/a	Confirmed	
☐	☒	The exact sample size (*n*) for each experimental group/condition, given as a discrete number and unit of measurement
☐	☒	A statement on whether measurements were taken from distinct samples or whether the same sample was measured repeatedly
☐	☒	The statistical test(s) used AND whether they are one- or two-sided<br>*Only common tests should be described solely by name; describe more complex techniques in the Methods section.*
☐	☒	A description of all covariates tested
☐	☒	A description of any assumptions or corrections, such as tests of normality and adjustment for multiple comparisons
☐	☒	A full description of the statistical parameters including central tendency (e.g. means) or other basic estimates (e.g. regression coefficient) AND variation (e.g. standard deviation) or associated estimates of uncertainty (e.g. confidence intervals)
☒	☐	For null hypothesis testing, the test statistic (e.g. *F*, *t*, *r*) with confidence intervals, effect sizes, degrees of freedom and *P* value noted<br>*Give P values as exact values whenever suitable.*
☒	☐	For Bayesian analysis, information on the choice of priors and Markov chain Monte Carlo settings
☒	☐	For hierarchical and complex designs, identification of the appropriate level for tests and full reporting of outcomes
☒	☐	Estimates of effect sizes (e.g. Cohen's *d*, Pearson's *r*), indicating how they were calculated

Our web collection on statistics for biologists contains articles on many of the points above.

Software and code

Policy information about availability of computer code

Data collection	The sequences of genes were downloaded from national center for biotechnology information (NCBI).<br>The information of proteins was obtained from UniProt.
Data analysis	Alignments of amino acid sequences were performed by Clustal Omega and ESPript 3.0.<br>Theoretical molecular weight and isoelectric point (pI) were predicted using Expasy.<br>The PDB strcutures of proteins were obtained using Phyre2.<br>t-test was used for statistical analyses.

For manuscripts utilizing custom algorithms or software that are central to the research but not yet described in published literature, software must be made available to editors and reviewers. We strongly encourage code deposition in a community repository (e.g. GitHub). See the Nature Portfolio guidelines for submitting code & software for further information.

Data

Policy information about availability of data

All manuscripts must include a data availability statement. This statement should provide the following information, where applicable:

- Accession codes, unique identifiers, or web links for publicly available datasets
- A description of any restrictions on data availability
- For clinical datasets or third party data, please ensure that the statement adheres to our policy

The mass spectrometry data has been deposited in PRIDE (https://www.ebi.ac.uk/pride/) with identifier PXD047656 for Fig. 2a-b, and identifier PXD048313 for Fig. 1a.

Research involving human participants, their data, or biological material

Policy information about studies with human participants or human data. See also policy information about sex, gender (identity/presentation), and sexual orientation and race, ethnicity and racism.

Reporting on sex and gender	n/a
Reporting on race, ethnicity, or other socially relevant groupings	n/a
Population characteristics	n/a
Recruitment	n/a
Ethics oversight	n/a

Note that full information on the approval of the study protocol must also be provided in the manuscript.

Field-specific reporting

Please select the one below that is the best fit for your research. If you are not sure, read the appropriate sections before making your selection.

☒ Life sciences ☐ Behavioural & social sciences ☐ Ecological, evolutionary & environmental sciences

For a reference copy of the document with all sections, see nature.com/documents/nr-reporting-summary-flat.pdf

Life sciences study design

All studies must disclose on these points even when the disclosure is negative.

Sample size	For bacterial colonization in turbots, 10 turbots were used per group.<br>For survival curves of zebrafish, 20 zebrafish was used per group.
Data exclusions	There were no data exclusions.
Replication	All experiments have been repeated for at least three times to ensure reproducibility. All attempts at replication in this study are successful.
Randomization	Animals were randomly selected into an infection group.
Blinding	The investigators were not blinded in this study.

Reporting for specific materials, systems and methods

We require information from authors about some types of materials, experimental systems and methods used in many studies. Here, indicate whether each material, system or method listed is relevant to your study. If you are not sure if a list item applies to your research, read the appropriate section before selecting a response.

Materials & experimental systems

n/a	Involved in the study
☐	☒ Antibodies
☐	☒ Eukaryotic cell lines
☒	☐ Palaeontology and archaeology
☐	☒ Animals and other organisms
☒	☐ Clinical data
☒	☐ Dual use research of concern
☒	☐ Plants

Methods

n/a	Involved in the study
☒	☐ ChIP-seq
☒	☐ Flow cytometry
☒	☐ MRI-based neuroimaging

Antibodies

Antibodies used	Rabbit monoclonal anti β-tubulin (Beyotime, Shanghai, China) (CAT# AF1216)<br>Rabbit monoclonal anti Lamin B1 (Beyotime, Shanghai, China) (CAT# AF1408)<br>Rabbit monoclonal anti AIF (Beyotime, Shanghai, China) (CAT# AF1273)<br>Mouse monoclonal anti Flag-tag (Beyotime, Shanghai, China) (CAT# AF519)<br>Goat anti-rabbit IgG-HRP (Beyotime, Shanghai, China) (CAT# A0208)<br>Goat anti-mouse IgG-HRP (Beyotime, Shanghai, China) (CAT# A0216)<br>Mouse monoclonal anti HA-tag (YEASEN, Shanghai, China) (CAT# 30701ES20)<br>Rabbit monoclonal anti γH2AX (Abcam, Cambridge, USA) (CAT# ab81299)<br>Rabbit monoclonal anti MIF (Abcam, Cambridge, USA) (CAT# ab176565)<br>Rabbit monoclonal anti PARP1 (Abcam, Cambridge, USA) (CAT# ab191217)<br>Rabbit polyclonal anti RpoA (HUABIO, Hangzhou, China) (Custom made)<br>Rabbit polyclonal anti EseB (HUABIO, Hangzhou, China) (Custom made)<br>Rabbit polyclonal anti EvpP (HUABIO, Hangzhou, China) (Custom made)<br>Mouse monoclonal anti PAR, clone 10H (Merck, Darmstadt, Germany) (CAT# MABC547)<br>Mouse monoclonal anti His-tag (Beyotime, Shanghai, China) (CAT# AH367)<br>Mouse monoclonal anti S-tag (Beyotime, Shanghai, China) (CAT# AF2930)
Validation	The commercial antibodies used were validated by the manufacturers. The antibody prepared in this study was validated by Western blot analysis.

Eukaryotic cell lines

Policy information about cell lines and Sex and Gender in Research

Cell line source(s)	HEK293T, HeLa, and J774A.1 cells were obtained from the American Type Culture Collection (ATCC).<br>HEK293T (ATCC CRL-11268)<br>HeLa (ATCC No.CCL2)<br>J774A.1 (ATCC TIB-67)<br>HeLa-Mock, HeLa-YfiD-HA, J774A.1-Mock, and J774A.1-YfiD-HA cells were engineered in this study.
Authentication	The identity of the cell lines was frequently checked by their morphological features.
Mycoplasma contamination	All cell lines were tested to be mycoplasma-negative by the standard PCR method.
Commonly misidentified lines (See ICLAC register)	No commonly misidentified cell lines are used in this study.

Animals and other research organisms

Policy information about studies involving animals; ARRIVE guidelines recommended for reporting animal research, and Sex and Gender in Research

Laboratory animals	Turbot (Scophthalmus maximus), 30 g<br>Zebrafish (Danio rerio), 3 cm
Wild animals	No wild animals were used in this study.
Reporting on sex	Sex was not considered in this study.
Field-collected samples	No field-collected samples were involved in this study.
Ethics oversight	All animal procedures performed were authorized by the animal care committee of the East China University of Science and Technology (2006272). The Experimental Animal Care and Use Guidelines from the Ministry of Science and Technology of China (MOST-2011-02) were rigorously adhered to.

Note that full information on the approval of the study protocol must also be provided in the manuscript.
